# Lung diffusing capacity for nitric oxide in space: microgravity gas density interactions

**DOI:** 10.3389/fphys.2023.1161062

**Published:** 2023-05-09

**Authors:** Lars L. Karlsson, Alain Van Muylem, Dag Linnarsson

**Affiliations:** ^1^ Department of Physiology and Pharmacology, Karolinska Institutet, Stockholm, Sweden; ^2^ Chest Department, Erasme University Hospital, Brussels, Belgium

**Keywords:** NO, gas density, diffusivity, microgravity, hypobaria, nitric oxide, space

## Abstract

**Introduction:** During manned space exploration lung health is threatened by toxic planetary dust and radiation. Thus, tests such as lung diffusing capacity (DL) are likely be used in planetary habitats to monitor lung health. During a DL maneuver the rate of uptake of an inspired blood-soluble gas such as nitric oxide (NO) is determined (DL_NO_). The aim of this study was to investigate the influence of altered gravity and reduced atmospheric pressure on the test results, since the atmospheric pressure in a habitat on the moon or on Mars is planned to be lower than on Earth. Changes of gravity are known to alter the blood filling of the lungs which in turn may modify the rate of gas uptake into the blood, and changes of atmospheric pressure may alter the speed of gas transport in the gas phase.

**Methods:** DL_NO_ was determined in 11 subjects on the ground and in microgravity on the International Space Station. Experiments were performed at both normal (1.0 atm absolute, ata) and reduced (0.7 ata) atmospheric pressures.

**Results:** On the ground, DL_NO_ did not differ between pressures, but in microgravity DL_NO_ was increased by 9.8% (9.5) (mean [SD]) and 18.3% (15.8) at 1.0 and 0.7 ata respectively, compared to normal gravity, 1.0 ata. There was a significant interaction between pressure and gravity (*p* = 0.0135).

**Discussion:** Estimates of the membrane (Dm_NO_) and gas phase (Dg_NO_) components of DL_NO_ suggested that at normal gravity a reduced pressure led to opposing effects in convective and diffusive transport in the gas phase, with no net effect of pressure. In contrast, a DL_NO_ increase with reduced pressure at microgravity is compatible with a substantial increase of Dm_NO_ partially offset by reduced Dg_NO_, the latter being compatible with interstitial edema. In microgravity therefore, Dm_NO_ would be proportionally underestimated from DL_NO_. We also conclude that normal values for DL in anticipation of planetary exploration should be determined not only on the ground but also at the gravity and pressure conditions of a future planetary habitat.

## 1 Introduction

Data from the manned space programs executed so far have shown that microgravity (µG, weightlessness) *per se* and in-flight activities such as extravehicular activity (space walks) do not appear to have negative impacts on lung function (Prisk et al., 2005; Prisk et al., 2006; Prisk et al., 2008; Karlsson et al., 2009). There are, however, other potential challenges of lung health during future exploratory space flights. These include the inhalation of toxic planetary dust (Linnarsson et al., 2012), and particles generated accidentally from onboard processes. Further, galactic cosmic and solar radiation may have a negative impact on the lung, analogous to that suggested for cardiovascular function (Delp et al., 2016; Hughson et al., 2016). Therefore, it would be justified to monitor lung health during long-term space flight using a battery of tests including spirometry, lung diffusing capacity (DL), and markers for inflammatory airway disease such as exhaled nitric oxide (NO). We therefore determined DL during a 6-month stay at the International Space Station (ISS). In preparation for future planetary exploration, we performed DL measurements at both normal and reduced cabin pressure since the atmospheric pressure in future planetary habitats is likely to be lower than normal to facilitate extravehicular activity (NASA, 2010). Moreover, we determined DL for NO (DL_NO_) rather than the traditionally used carbon monoxide (DL_CO_), as the uptake of endogenously formed NO from the lung periphery to the blood may potentially influence the results of tests of exhaled NO.

The lung tissue is exquisitely sensitive to alterations of gravity due to its structure, with millions of air spaces separated by thin, blood-filled elastic septa (West, 2012). This normally results in gravity-induced gradients of tissue density, alveolar size, alveolar ventilation, lung-capillary blood filling, and perfusion. In µG, such gradients will disappear, resulting in more homogeneous distributions of the above as shown in a series of studies on lung physiology that were performed on the US Space Shuttle during the 1990s (Prisk et al., 1993; Prisk et al., 1994; Elliott et al., 1994; Elliott et al., 1996; Guy et al., 1994; Verbanck et al., 1996; Verbanck et al., 1997). Non-invasive estimates of regional distribution of ventilation (Guy et al., 1994; Verbanck et al., 1996) and perfusion (Prisk et al., 1994) in the lungs confirmed that these distributions became more homogeneous than at normal gravity during sustained (1–2 weeks) microgravity. The efficiency of the pulmonary gas exchange was studied further, with measurements of DL_CO_ (Prisk et al., 1993; Verbanck et al., 1997). Such measurements were of particular interest, because an expected sustained increased intrathoracic blood filling in microgravity compared to upright posture in normal gravity could either improve DL_CO_ by creating a larger blood/gas interface or impair DL_CO_ by causing an interstitial edema (Permutt, 1967). As DL_CO_ depends on both the conductance through alveolo-capillary membrane (Dm) and the lung-capillary blood volume (Roughton and Forster, 1957), Prisk et al. (1993) estimated both these parameters. Using a technique involving breath holding at vital capacity, they found that both the membrane component of DL_CO_ (Dm_CO_) and the lung-capillary blood volume were increased. They concluded that the increased Dm_CO_ was likely due to an expanded blood-gas interface area, in turn resulting from a more homogeneous filling of the lung capillaries throughout the lung compared to normal gravity. Both a 28% increase of DL_CO_, and a 27% increase of Dm_CO_ persisted over 9 days in microgravity. Verbanck et al. (1997) observed a similar increase after 2–9 days of microgravity, using a rebreathing technique to determine DL_CO_.

Corresponding data for DL and Dm for long duration space flight have so far not been available, and it cannot be assumed *a priori* that findings from the first one or 2 weeks of microgravity will be representative for the duration of a 6-month stay at the ISS, or for longer periods during future planetary exploration and in reduced atmospheric pressure. Earlier work from our laboratory has demonstrated that DL_NO_ is influenced by ambient pressure (Linnarsson et al., 2013); a four-fold increase in pressure and gas density reduces DL_NO_ by slowing diffusive gas transport in the lung periphery. In support of the same notion, Nie et al. (2001) showed that reducing the density of the breathing gas by a factor of three with helium led to signs of enhanced uptake of inhaled NO to the pulmonary circulation. In contrast, reducing the ambient pressure and the gas density from that at sea level to 50% of normal had only a marginal effect on DL_NO_ (Linnarsson et al., 2013). Similarly, Gray et al. (1986) showed that when DL_CO_ was determined in normoxia at 70% of normal pressure and gas density, it did not differ from that observed at sea level. In normal gravity therefore, it appears as the levels of pressure and gas density anticipated in future planetary habitats are not low enough to significantly influence DL_NO_ and DL_CO_.

This will not necessarily be the case in microgravity: the results of earlier work on diffusion-convection interaction in the lungs during sustained microgravity (Prisk et al., 1996; Lauzon et al., 1997) have shown that gas density alters diffusion-convection dependent ventilation inhomogeneity differently between normal gravity and microgravity. Single breath washout maneuvers were studied, where the background gas had normal density, and low concentrations of helium and sulfur hexafluoride (SF_6_) were added as tracer gases. The ratios between the slopes of phase III of the gases were determined, and the disparities in this parameter between the two gravity conditions were suggested to depend on differences in cardiogenic mixing of gas at an acinar level. These results are not easily translated to the present experimental design, in which the densities of the background gas and the tracer gas (in our case NO) were changed in parallel. Therefore, a conservative prediction for the present experiments must be that diffusion-convection dependent ventilation inhomogeneity will differ between gravity conditions. As a consequence, a change in gas density is likely to alter peripheral gas transport differently between normal gravity and microgravity, potentially influencing DL_NO_.

We hypothesized further that with normal gas density, a microgravity induced increase of DL_NO_ will persist, as previously observed for DL_CO_ during the first weeks in space due to a continued expanded blood-gas interface. An expected reduction of the overall blood volume with time (Alfrey et al., 1996) would not be expected to modify DL_NO_ proportionally, since, in contrast to DL_CO_, DL_NO_ is not sensitive to the amount of blood in the lung-capillary blood (Johnson et al., 1996; Zavorsky et al., 2008) but merely to its distribution, which is not necessarily altered proportionally. However, any development of interstitial edema with time should attenuate the expected increase of DL_NO_ in microgravity by thickening the alveolo-capillary membrane and/or narrowing peripheral airways (Guy et al., 1991; Elliott et al., 1994).

## 2 Methods

### 2.1 Subjects and ethical approval

The research was approved by Etikprövningsmyndigheten, The Swedish Ethical Review Authority, nr 2009/1341—31/3. Corresponding approvals were obtained from the internal review boards of the European Space Agency (ESA), the US National Aeronautics and Space Administration (NASA) and the Japanese Space Agency (JAXA). The subjects received both oral and written information about the procedures and their scientific background, and written consents were obtained, and the subjects were aware of their right to withdraw from the experiment without prejudice at any time.

### 2.2 Experimental conditions

The present experiments were performed in parallel with a study on exhaled NO, which will be reported elsewhere. Experiments in normal gravity [1G, Baseline data collection (BDC)] mostly took place at the Johnson Space Center, Houston, TX, United States, with two trials at the European Astronaut Centre, Cologne, Germany and one at Skrydsrup Air Force Base, Denmark. Procedures were performed at normal atmospheric pressure [1.0 atmosphere absolute (ata)] and at 0.7 ata in hypobaric chambers, which is equivalent to an altitude of 3,000 m (10,000 ft). The experiments in weightlessness (microgravity, µG) were performed on the ISS, at normal pressure and at 0.7 ata, with the latter in the US Air Lock that normally is used for preparations before extravehicular activity. The cabin atmosphere during the 1.0 ata experiments was air, and at 0.7 ata was a mixture of 27.5% oxygen in nitrogen. This percentage was a compromise between the desire to avoid hypoxia and concurrently maintain the oxygen fractional concentration below the threshold specified for certain equipment in the US Air Lock. The resulting inspired oxygen partial pressure (PO_2_) in both gravity conditions, at 0.7 ata was 19.3 kPa (146 mmHg), corresponding to an altitude of 700 m (2,300 ft) if breathing air. At 1.0 ata, PO_2_ averaged 21.2 kPa (159 mmHg) at 1G and 21,0 kPa (158 mmHg) in µG. Temperature and relative humidity averaged 22.8°C and 66% during BDC. Corresponding inflight values were 24.1°C and 42%.

### 2.3 Instrumentation and measurements

**FIGURE 1:**
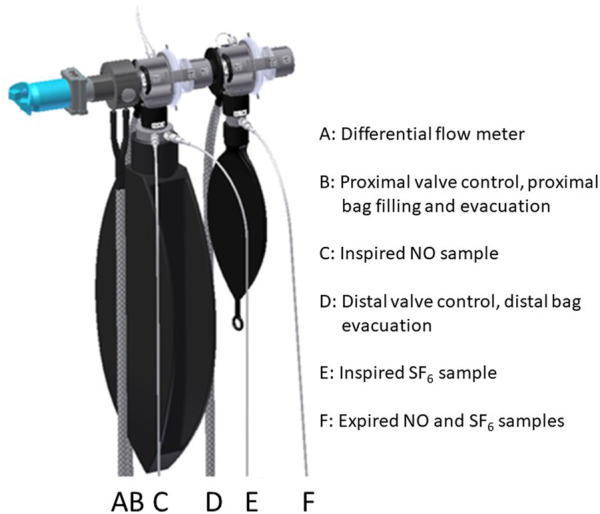
Respiratory valve unit. RVU.

Dedicated instrumentation was developed for the European Space Agency by the Danish Aerospace Corporation, Odense, Denmark. Key components in the system included a Portable Pulmonary Function System, a respiratory valve unit (RVU, Figure 1) and a commercially available NO analyzer (Niox Mino, Aerocrine, Sweden and Circassa, United Kingdom). The NO analyzer required 100 s to analyze a gas sample, so the system for gas sampling necessarily became more complex than that used in a previous study (Linnarsson et al., 2013), where rapid-responding gas analyzers permitted on-line analysis of respired gases. The system provided sensors for respiratory flow, mouthpiece pressure, sulfur hexafluoride (SF_6_) and metabolic gases. The RVU (Figure 1) permitted inhalation of a vital capacity of premixed gas from a 10-L bag and sampling of exhaled gas during a specific volume interval in a 1-L bag (small bag) for subsequent analysis. A pneumatic system and a set of solenoid valves controlled the two three-way valves of the RVU, the sampling of gas for analysis, the evacuation of the bags between measurement sequences, and the filling of the 10-L bag. The Portable Pulmonary Function System also included a system for pre-programmed experimental sequencing, in which the astronauts were given step-by-step instructions on a screen regarding set up, calibration and the timing of breathing maneuvers. The investigators and personnel from Danish Aerospace Corporation monitored the experiments on site during BDC and on-line during inflight experiment.

### 2.4 Diffusing capacity measurement procedure

**FIGURE 2:**
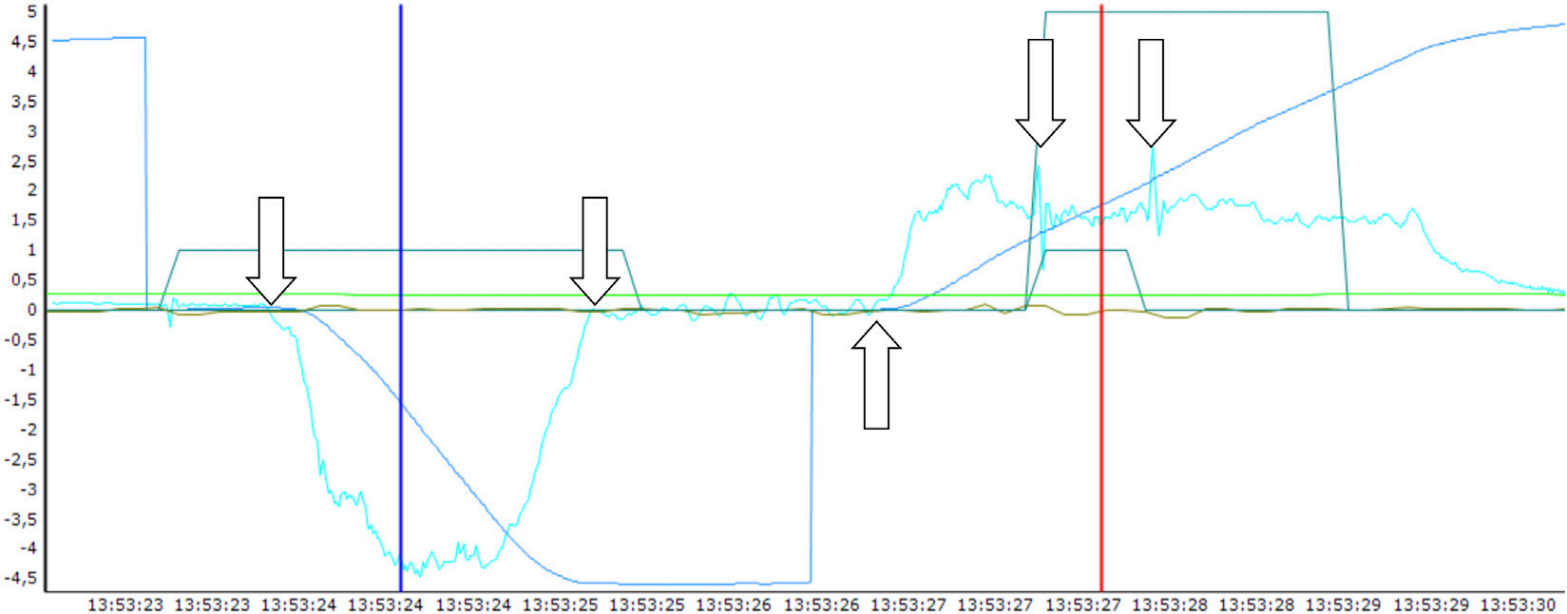
The breathing maneuver for determination of lung diffusing capacity for NO is exemplified in this screenshot from the data analysis. The light blue tracing is flow (L/s) and the dark blue tracing is volume (L) with the scale for both shown to the left. The low, trapezoid, dark green tracing shows the control signal for respiratory valve action. The remaining tracings are not relevant for the present step of the analysis. The arrows indicate the following time points (starting left): 1. Start of rapid full inspiration from the big bag containing NO and SF6 as tracer gases; 2. Start of breath holding; 3. Start of a controlled expiration; 4 and 5. Start and end respectively, of sampling of expired gas into the small bag. The blue cursor shows the start and the red cursor the end of the estimated period for NO uptake into the blood. The gas contents of the big bag had been analyzed before the inspiration, and the corresponding contents of the small bag were analyzed after the end of the maneuver.

DL_NO_ was determined according to clinical standards for DL_CO_ (Macintyre et al., 2005) and modified according to Zavorsky et al. (2008), with a shorter breath-hold time for NO. Experimental sessions were started with calibration of the differential pressure flowmeter using a 3-L syringe as described by Yeh et al. (1982). Gas sensor linearity had been determined earlier, and since the subsequent data analysis was based on ratios between concentrations, the absolute readings of the gas sensors were not calibrated on each occasion. The subject initiated the measurement sequence, with the first step filling the 10-L bag with an individually preset volume of air containing 1% SF6. In parallel, a small volume of a second premixed gas containing 800 ppm NO in nitrogen was added so to receive a final NO concentration of 1 ppm in the bag, which was agitated to promote mixing. *In situ* mixing was necessary, otherwise NO and oxygen would generate toxic byproducts if stored together longer than a few minutes. Thereafter, gas from the same bag was sampled to the NO and SF_6_ analyzers to determine the inspired concentrations. Wearing a nose clip, the subject then donned the RVU mouthpiece and took 3–4 breaths of tidal breathing after which they: 1) performed a maximal expiration to residual volume; 2) activated the continued measurement sequence as they were connected to the 10-L bag; 3) inspired a vital capacity as rapidly as possible; 4) held their breath for 2–3 s while the valve to the 10-L bag was switched and; 5) exhaled at a rate of 2 L/s into the cabin air, guided by visual feed-back on a screen. After 1 L of gas had been exhaled, the second valve of the RVU switched to fill the 1-L bag and then switched back when 2 L of gas had been expired. The test sequence ended with sampling of gas from the 1-L bag for analysis of NO and SF_6_. In the data analysis this sample was considered to represent alveolar gas at a point in time half-way through the filling of the 1-L bag. Figure 2 shows a typical recording of respiratory flow and volume.

### 2.5 Data analysis

The lung diffusing capacity of NO (DL_NO_ in ml∙min^−1^∙mmHg^−1^) was calculated according to current standards (Macintyre et al., 2005). An anatomical dead space of body mass in kg*2.2 was assumed and the instrument dead space was 100 mL. The breath-hold duration was determined from the flow and mouthpiece pressure recordings which permitted estimation of the point in time for the arrival of the inspired gas to the alveoli and the timing of the alveolar sample respectively, the latter since there was a transient drop in expiratory flow resistance during bag filling (Figure 2). The alveolar volume (VA, L BTPS) and KNO, the ratio between DL_NO_ and VA, were also obtained for each DL_NO_ measurement.

### 2.6 Statistics

Data were analyzed by mixed linear models. DL_NO_, VA and KNO were analyzed as factorial experiments with the effect of gravity, pressure, and gravity-pressure interaction. The correlation between measurements was set to unstructured. The conditions for the analysis were controlled by residual analysis.

## 3 Results

A group of 11 astronauts were studied, eight males and three females. Their age, height and body mass ranged from 37 to 51 years, 1.65–1.86 m and 63–92 kg, respectively. Each astronaut participated in between 2 and 4 1G sessions during the pre-flight period and 10 out of 11 in 2 µG sessions on the ISS, whereas one subject only had 1 µG session. The first and last 1G sessions occurred during days −191 (77) [mean (SD)] and −86 (22) before launch. The corresponding days for the µG sessions were +36 (16) and +103 (32) after the launch. Of the 2 µG sessions, one was performed at 1.0 ata only and one at both atmospheric pressures.

Complete data sets from all combinations of gravity and pressure were obtained in nine subjects. For the two first subjects studied in microgravity, the electrochemical sensing element of the NO analyzer failed after decompression to 0.7 ata, so data could then be obtained only at normal pressure.

**FIGURE 3:**
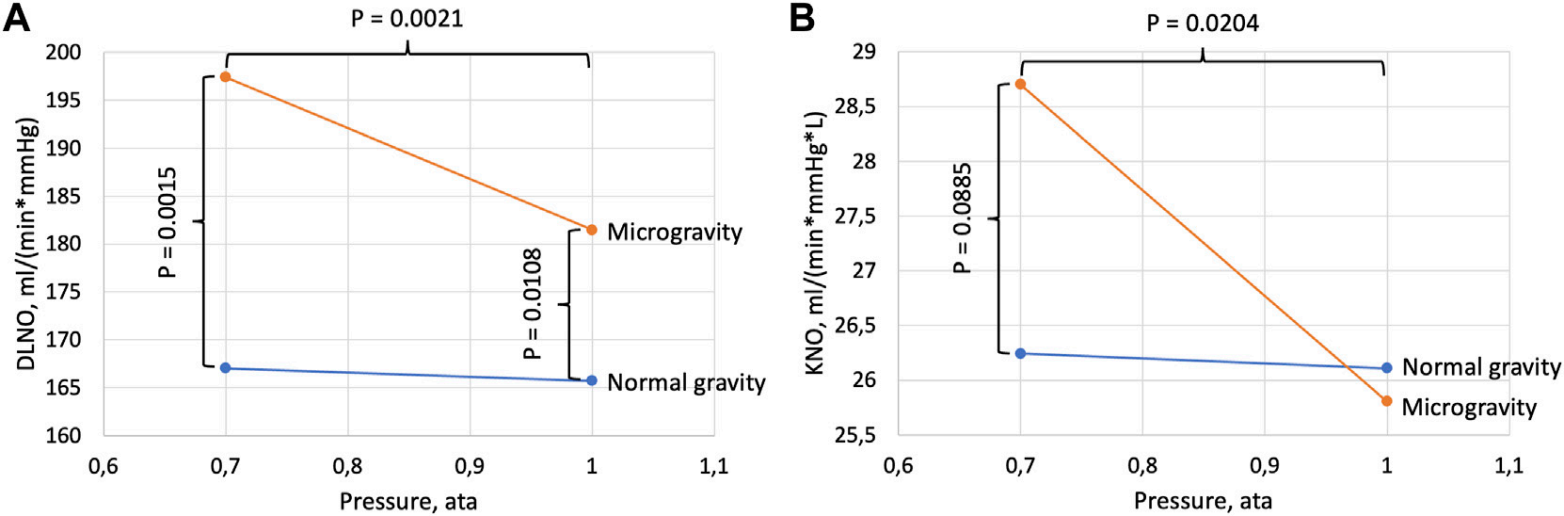
Diffusing capacity for NO (DL_NO_, panel **(A)**) and DLNO normalized for alveolar volume (KNO, panel **(B)**). Data are shown as a function of cabin pressure. *p* values refer to differences between pressure conditions within one gravity condition (horizontal brackets) and differences between gravity conditions within one pressure condition (vertical brackets). In normal gravity there were no significant effects of pressure for DLNO (*p* = 0.4198) or KNO (*p* = 0.6677).

Group mean results are shown in Table 1 and Figure 3A, B. There were no significant time trends within the BDC and inflight periods respectively. Therefore, data are mean values for all measurements during each of the two periods.

**TABLE 1:** Group mean values and standard deviations of lung diffusing capacity for NO (DL_NO_), alveolar volume (VA) and the ratio DL_NO_/VA (KNO) during four combinations of gravity and pressure (atmospheres absolute, ata). *p* values in the three right-hand columns refer to effects of gravity (G), pressure (P), and gravity-pressure interactions, respectively.

Variable	G level, g	Pressure, ata	Mean	SD	G effect	P Effect	G * P
DL_NO_, ml/(min*mmHg)	1	1.0	165.7	22.9	0.0108	0.4198	0.0135
		0.7	167.0	23.2			
	0	1.0	181.5	26.3			
		0.7	197.4	29.8			
VA, L_BTPS_	1	1.0	6.372	0.874	0.0001	0.8247	0.3109
		0.7	6.401	0.948			
	0	1.0	7.063	0.971			
		0.7	6.922	0.959			
KNO ml/(min*mmHg*L)	1	1.0	26.11	2.38	0.6687	0.6677	0.0339
		0.7	26.24	2.63			
	0	1.0	25.81	2.71			
		0.7	28.70	3.72			

There was a significant effect of gravity on DL_NO_ (Table 1) with a likewise significant gravity-pressure interaction, and DL_NO_ at 1.0 ata was increased by 10% in microgravity compared to normal gravity (Figure 2). The corresponding increase at 0.7 ata was 18%. Generally, the alveolar volumes were increased in microgravity (11% and 8% respectively at 1.0 and 0.7 ata) with no difference between pressures. DL_NO_ normalized for alveolar volume (DL_NO_/VA = KNO) did not differ between gravity conditions at 1.0 ata but tended to be increased in microgravity at 0.7 ata. In microgravity, KNO was 11% larger at 0.7 ata than at 1.0 ta.

## 4 Discussion

The main finding of the present study is an interaction between gravity and pressure effects on the rate of lung gas-to-blood transfer as determined with DL_NO_. Thus, we observed an increase of DL_NO_ in microgravity compared to normal gravity, and a further increase with a combination of microgravity and reduced atmospheric pressure. Concurrently, there was no effect of pressure in normal gravity.

### 4.1 Effects of gravity at normal pressure

To determine DL, we used the same type of maneuver as Prisk et al. (1993), namely, a breath-hold made with an open glottis but found a microgravity-related increase that was only 34% of their observations. The most likely explanation for this difference could be timing: Prisk et al. (1993) obtained their results during the first 9 days of microgravity, whereas in the present study, test days ranged between Day 15 and Day 254 with an average of Day 87. It can be envisaged that there would be an initial distention of the pulmonary circulation, which would then be gradually reduced as total blood volume is downregulated by some 15% during the first weeks in space (Alfrey et al., 1996). A corresponding reduction of the pulmonary-capillary blood volume and an associated reduction of the area of the blood-gas interface is a possible contributing factor. However, data from Norsk et al. (2015) suggest that a hyperkinetic blood circulation persists after 3–6 months in space, with cardiac output at rest being elevated by 35%–41% compared to sitting control in normal gravity. Norsk et al. (2015) also found a proportionally reduced total peripheral resistance, suggesting systemic vasodilation as the principal mechanism for the hyperkinesia, but at the same time included a maintained increase of the central blood volume as a contributing factor. Several other observations also point to a continued cranial blood and fluid displacement during long-term space flight, such as facial edema and the Spaceflight Associated Neuro-ocular Syndrome (Mader et al., 2011; Ly et al., 2022). In summary, our finding of a 10% increase of DL_NO_ in microgravity at normal pressure is less than would be expected, based on previous continued hyperkinetic circulation and cranial redistribution of blood data. However, the cranial redistribution of tissue fluid may have counteracted partly the effect of an enlarged blood-gas interface by causing an interstitial edema (Permutt, 1967), narrowing the diffusion path in the lung periphery. The findings by Prisk et al. (1993) and Verbanck et al. (1997) suggest that such an impediment to alveolar-to-blood transfer in the lungs had little impact during the first weeks in microgravity, whereas the present data are compatible with a development of such an impediment over time.

The combination of increases in DL_NO_ and VA of similar magnitude is illustrated by the unchanged KNO value when comparing preflight and inflight data obtained at 1.0 ata (Figure 2, right panel). Miserocchi et al. (2008) studied the relationship between Dm_CO_ and VA, demonstrating a positive relationship. Their mathematical model included alveolar expansion by both unfolding and stretching and suggested that stretching, which led to a thinning of the membrane, dominated at lung volumes larger than 80% of total lung capacity. As the present DL_NO_ maneuvers and those of Prisk et al. (1993) included breath holding after a full inspiration, the 10% increase of VA in microgravity in our study and the corresponding 5% increase reported by Prisk et al. (1993) should have contributed to the observed DL increases, but clearly to a much smaller extent in the former case than seen in the present data following long-term microgravity.

### 4.2 Effects of pressure at normal gravity

Our observations that DL_NO_ and KNO did not differ between pressure conditions at normal gravity are in agreement with previous work showing that a 30%–50% reduction of pressure did not significantly enhance DL (Gray et al., 1986; Linnarsson et al., 2013).

### 4.3 Combined effects of pressure and gravity

In microgravity, DL_NO_ and KNO were increased by 9% and 11% at 0.7 ata and 1.0 ata respectively, suggesting a net enhancement of diffusive transport in the gas phase in the lung periphery at the lower pressure. The present data can be analyzed further, as described previously (Linnarsson et al., 2013). The classical equation describing the components of the resistance (=1/conductivity) for gas transport in the lungs during a DL_CO_ maneuver (Roughton and Forster, 1957) can be rewritten for DL_NO_ as:

$$1/\;{DL}_{NO}=1/\;{Dg}_{NO}+1/\;{Dm}_{NO}$$ (1)

where Dg is the conductivity for NO in the gas phase.

Corresponding equations for DL_CO_ considered the Dg term negligible, but instead had a term related to the pulmonary-capillary blood volume, which can be neglected for DL_NO_ (Zavorsky et al., 2008). The diffusivity of a tracer gas such as NO is inversely proportional to the density and pressure of the background gas. Assuming that this is true also for the net effect of diffusive and convective NO transport in the gas phase, Eq. 1 can be rewritten as:

$$1/{DL}_{NO}=\left(1/\;{Dg}_{NO}\right)\;*\;\left(P_{1}/P_{0}\right)+1/\;{Dm}_{NO}$$ (2)

Where P_1_ is the pressure at the time of measurement and P_0_ is a reference pressure at which Dg is defined.

**FIGURE 4:**
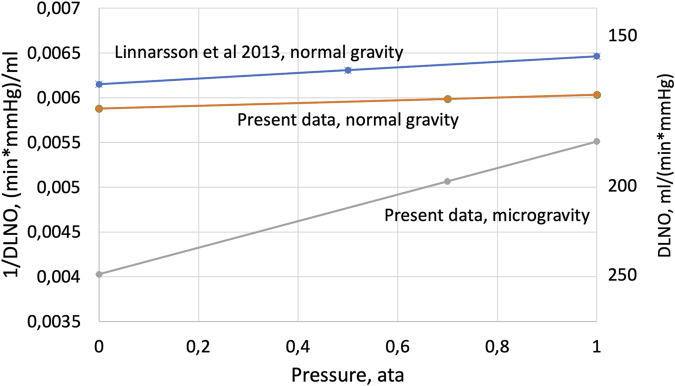
The inverse value of DL_NO_ plotted as a function of cabin pressure according to Eq. 2—Experimental data from the range 1 ata to 0.5 or 0.7 ata have been extrapolated to a theoretical situation at 0 ata, where transport in the gas phase would be infinitely rapid, i.e., no impact of density on NO transport in the gas phase. Under such assumptions, the intercept with the Y-axis represents the inverse of the true membrane component of DL_NO_.

The equation has the form of a linear relationship in which 1/(Dg_NO_ * *P*_*0*_) is the slope and 1/Dm_NO_ the intercept with the Y-axis. The present DL_NO_ data are shown accordingly in Figure 4 together with earlier data from our laboratory (Linnarsson et al., 2013), and in Table 2.

**TABLE 2:** Estimates of the membrane component (Dm) and the gas phase component (Dg) of the lung diffusing coefficient for NO [ml/(min*mmHg)] as obtained from Eq. 2 and as shown in Figure 4.

	Dm	1/Dm	Dg	1/Dg
1G	170.1	0.00588	6,386	0.00016
µG	248.4	0.00403	674	0.00148

As illustrated in Figure 4, data from normal gravity show a modest, if any, net effect of pressure on DL_NO_ in the pressure range below 1.0 ata. As already discussed above, any effects of pressure on DL_NO_ are likely to be the result of combined effects on diffusive and convective gas transport, resulting in a very small net 1/Dg component in normal gravity (Table 2); thus, DL_NO_ as determined at 1.0 ata then closely approximates to Dm_NO_. This contrasts with DL_NO_ changes with pressure in microgravity; the analysis as shown in Figure 4 and Table 2 suggests that the 1/Dg component then is an order of magnitude larger than at normal gravity. The much higher resistance (1/Dg) and lower conductivity (Dg) for NO transport in the gas phase in microgravity are compatible with, but not proof of, a narrowing of the diffusion path for NO and interstitial edema in peripheral airways. Concurrently, our simplified model as described in Eq. 2; Figure 4 suggests that Dm in microgravity becomes substantially underestimated by DL_NO_ when determined at 1.0 ata, so that without the Dg component, the true underlying Dm value would have been 248 mL/(min * mmHg). This is 37% more than the DL_NO_ value determined in microgravity at 1.0 ata, and 50% more than in normal gravity and pressure. Such data can only be reconciled with the notion of interstitial edema if the relative expansion of the surface area of blood-gas interface is substantially larger than a coexisting increase of its thickness (Prisk et al., 1993).

## 5 Limitations

### 5.1 Experimental conditions

Our initial goal was to perform studies at 0.5 ata, which was the cabin pressure foreseen in future planetary habitats (NASA, 2010). This atmosphere was proposed to have an oxygen concentration of 32%, resulting in a hypoxic environment generally accepted in commercial aircrafts and equivalent to air breathing at 3,000 m (10,000 ft) altitude. The discovery of Spaceflight Associated Neuro-ocular Syndrome (Mader et al., 2011, then named Visual Impairment and Intracranial Pressure) however, led to concerns over hypoxia during space exploration causing worsening of a suspected elevation of intracranial pressure in astronauts with this syndrome. Accordingly, the present experiments were redesigned to avoid hypoxia, the minimum allowed pressure was changed from 0.5 to 0.7 ata, and the inspired oxygen percentage was set to 27.5%, which was the maximum allowed for the design of some of the equipment. Further experiments at the appropriate pressure and oxygen concentration are thus required for determination of how lung function tests such as DL_NO_ will be influenced by the combination of altered gravity and atmosphere in future planetary habitats.

An additional consequence of the above constraints was that the inspired PO_2_ was 1.8 kPa (13 mmHg) lower at 0.7 ata than at 1.0 ata. Theoretically hypoxia may influence DL_NO_ as well as DL_CO_ by increasing capillary recruitment, but the present reduction of PO_2_ at 0.7 ata is by far too small to have such and effect (Capen et al., 1981). Moreover, DL_NO_, in contrast to DL_CO_, is not sensitive to the O_2_ saturation of the hemoglobin in the pulmonary capillary blood (Zavorsky et al., 2008). In summary, therefore, we conclude that the small difference in inspired PO_2_ between the two pressure conditions had no impact on the results of our study.

### 5.2 Validity of the present model for NO transport in the gas phase

The present assumption that the net effect on diffusive and convective gas transport in the lung periphery is proportional to pressure is admittedly an oversimplification. Furthermore, extrapolation to 0.0 ata based on two points at 0.7 and 1.0 ata for the present data may rightly be questioned, but the similarity between the present normal gravity data and those from Linnarsson et al. (2013) lends support to the extrapolation. Justification for this assumption is based on the function from our previous work at normal gravity, using measurements at 0.5, 1.0 and 4.0 ata, the latter point not shown in Figure 4. Nevertheless, we believe that the simple model illustrated in Figure 4 provides rough estimates on the relative sizes of the resistances to gas transport through the gas phase and further through the alveolo-capillary membrane in microgravity.

## 6 Perspective

Lung diffusing capacity is a non-invasive test with a high clinical relevance for monitoring lung health. We show that it can be performed easily by trained crew members in a space flight environment; this is necessary, as baseline values obtained on the ground will not accurately reflect corresponding values in a habitat environment with reduced atmospheric pressure and gravity.

## Data Availability

The raw data supporting the conclusion of this article will be made available by the authors, without undue reservation.
